# The regulatory role and mechanism of energy metabolism and immune response in head and neck cancer

**DOI:** 10.1016/j.gendis.2025.101607

**Published:** 2025-03-19

**Authors:** Haofan Li, Qiu Peng, Linda Oyang, Wenjuan Yang, Shizhen Li, Yaqian Han, Mingjing Peng, Shiming Tan, Longzheng Xia, Jinguan Lin, Xuemeng Xu, Nayiyuan Wu, Yanyan Tang, Xia Luo, Xianjie Jiang, Qianjin Liao, Yujuan Zhou

**Affiliations:** a The Affiliated Cancer Hospital of Xiangya School of Medicine, Central South University/Hunan Cancer Hospital, Hunan Key Laboratory of Cancer Metabolism, Changsha, Hunan 410013, China; b Hengyang Medical School, University of South China, Hengyang, Hunan 421001, China; c Hunan Engineering Research Center of Tumor Organoid Technology and Application, Public Service Platform of Tumor Organoids Technology, Changsha, Hunan 410013, China; d Department of Oncology, Hunan Provincial People’ s Hospital (The first-affiliated hospital of Hunan normal university), 61 Jiefang West Road, Changsha, Hunan 410005, China

**Keywords:** Amino acid metabolism, Glucose metabolism, Head and neck cancer, Immune response, Lipid metabolism, Therapy

## Abstract

Head and neck cancer, which includes cancers of the mouth, larynx, and pharynx, is one of the six most common cancers worldwide. Common risk factors include smoking, excessive alcohol consumption, betel nut chewing, and viruses such as HPV and EBV. Tumor cells often exhibit distinct metabolic characteristics compared with normal cells, highlighting a key area for potential intervention. By targeting these metabolic pathways, it is possible to influence tumor initiation and progression. Therefore, this review primarily describes the alterations in glucose metabolism, amino acid metabolism, lipid metabolism, and the immune system in head and neck cancer patients and discusses potential treatment strategies to advance the understanding of head and neck cancer and the development of therapeutic drugs for it.

## Introduction

Head and neck cancer is a prevalent tumor worldwide, with an estimated 450,000 deaths and 890,000 new cases in 2022.^1^ Head and neck squamous cell carcinoma (HNSCC) is the most common subtype of head and neck cancer and often originates from the mucosal epithelium of the oral cavity, pharynx, and larynx.^2^^,^^3^ In the United States, the incidence rate among men is two to four times that of women, highlighting a significant gender disparity.^4^ Research has shown that the prognosis for patients with HNSCC has improved slightly over the past decade, a positive trend that has been attributed to the global decline in tobacco use. Despite these advances, however, the incidence of HNSCC has continued to increase, highlighting an ongoing challenge in cancer control and prevention.^5^, ^6^, ^7^ Otto Warburg first observed that cancer cells preferentially rely on glycolysis for energy production over oxidative phosphorylation, even under aerobic conditions, a phenomenon known as the Warburg effect.^8^ This discovery has led to extensive research into the metabolic reprogramming of tumors. Amino acids, as essential nutrients, not only serve as carbon and nitrogen sources for macromolecule biosynthesis but also function as intermediates entering the tricarboxylic acid cycle. Cancer cells exhibit a significantly higher consumption of amino acids compared with normal cells, with amino acids such as glutamine contributing nearly half of the carbon sources in cancer cells, while only approximately 10% originates from glucose.^9^ To target this metabolic dysregulation, researchers have adopted the inhibition of glutamine metabolism as a therapeutic strategy. For instance, l-asparaginase has been incorporated into the standard chemotherapy regimens for acute lymphoblastic leukemia.^10^ Furthermore, in addition to alterations in amino acid metabolism, cancer cells exploit lipid metabolism to support rapid proliferation, invasion, and metastasis.^11^ Lipids are not only a major energy source but also play a crucial role in the construction of biological membranes. Triglycerides are metabolized via β-oxidation to generate ATP, thereby fulfilling the high energy demands of proliferating cancer cells. Moreover, lipid metabolites act as second messengers involved in critical signal transduction pathways. Phospholipases generate molecules such as diacylglycerol and phosphatidic acid, which activate oncogenic pathways, including RAS, phosphoinositide 3-kinase (PI3K), and protein kinase C (PKC), thereby promoting tumor progression.^12^^,^^13^ These alterations result in a complex metabolic reprogramming of cancer cells, providing the energy and substances necessary for tumor cell growth and survival and providing potential targets for therapy.

Immune cells play a critical role in the progression of tumors. Immunity can be broadly divided into innate immunity and acquired immunity. The innate immune system is characterized by immune cells such as macrophages, neutrophils, monocytes, eosinophils, basophils, and natural killer cells.^14^ Acquired immunity, also known as adaptive immunity, emerges as the immunity produced by the body in response to stimulation by exogenous antigens, with lymphocytes typically including T cells and B cells playing a pivotal role. Furthermore, adaptive immunity exhibits properties of diversity, memorability, and specificity.^15^ Significantly, research indicates that the phenotype and function of immune cells are influenced by their metabolic changes. During the critical phase of tumor progression, the metabolism of both tumor cells and infiltrating immune cells changes, creating an anti-tumor or pro-tumor microenvironment.^16^ For instance, in the tumor microenvironment, the observed changes in glucose, lipid, and amino acid metabolism in macrophages dramatically affect tumor progression.^16^

In conclusion, this review aims to deeply investigate the interplay between energy metabolism alterations and immune responses in the specific context of HNSCC. Within this framework, it highlights the key molecular players and explores the therapeutic significance of metabolic reprogramming and the intricate field of immune processes, ultimately providing insights into potential approaches for therapeutic intervention in HNSCC.

## Roles of glucose metabolism in head and neck cancer

In human metabolism, glucose is broken down through the processes of glycolysis and oxidative phosphorylation. This provides fuel for physiological functions and generates intermediates for biosynthetic pathways such as nucleotide, protein, and lipid synthesis.^17^ In the tumor microenvironment, the supply of glucose and oxygen is limited. However, under hypoxic conditions, tumor tissue can generate ATP through glycolysis more efficiently than by oxidative phosphorylation, allowing for rapid growth.^18^, ^19^, ^20^, ^21^, ^22^, ^23^ It produces large amounts of lactic acid through glycolysis, which facilitates the formation of a tumor microenvironment that promotes tumor growth.^17^^,^^24^

The process of glucose metabolism involves several rate-limiting steps, with the transport of glucose across the cellular membrane being a crucial bottleneck.^25^ The glucose transporter proteins (GLUTs) are at the core of this process and help the cell to take up glucose.^26^ GLUT1, a major member of the GLUT family, plays a pivotal role in regulating glucose uptake in nearly all cells. The up-regulation of GLUT1 in cancer cells has been observed to promote the proliferation and invasion of cancer cells.^27^ Thus, GLUT1 represents a crucial rate-limiting factor in glucose metabolism.^28^^,^^29^ GLUT1 plays a pivotal role in the malignant progression of oral squamous cell carcinoma (OSCC), and its overexpression is associated with augmented proliferation and invasion of OSCC cells and poor prognosis.^30^, ^31^, ^32^ GLUT4 is aberrantly overexpressed in HNSCC, promotes the proliferation and invasion of cancer cells through the tripartite motif-containing 24 (TRIM24)-DDX58 axis, and is associated with poor prognosis.^33^ A study by Sun et al revealed the role of the gram-negative bacillus *Fusobacterium nucleatum* (*F. nucleatum*) as a significant pathogenic bacterium in tumorigenesis. This study showed that *F. nucleatum* activated the GalNAcAutophagy-TBC1D5 signaling axis, which promoted the aggregation of GLUT1 at the plasma membrane and glycolysis in OSCC patients, thereby fostering lactate accumulation.^34^ The up-regulation of kynurenic acid (KYNA) in cancer cells has been associated with tumor immune dysfunction and poorer survival prognosis in patients with OSCC. A study by Zhou et al demonstrated that *Streptococcus mutans* (*S. mutans*) stimulates the production of KYNA, which in turn promotes neutrophil expansion and interleukin (IL)-1β secretion. The release of IL-1β induces CD8^+^ T cell exhaustion and accelerates OSCC progression. Furthermore, KYNA reduces the therapeutic efficacy of programmed death-ligand 1 (PD-L1) and IL-1β inhibition in oral cancer models.^35^ In addition, circular RNAs (circRNAs) and long non-coding RNAs (lncRNAs) have been reported to play a regulatory role in cancer cell metastasis by modulating glucose metabolism. For instance, circRNA_100290 functions as a competing endogenous RNA (ceRNA) of miR-378, counteracting the inhibitory effects of miR-378a on GLUT1, promoting glycolysis and metastasis in OSCC.^36^ circATRNL1 expression is reduced in OSCC, while itself promotes radiosensitization.^37^ lnc-p23154 promotes glycolysis in OSCC cells, and it enhances GLUT1 expression through repressing miR-378a-3p transcription.^30^ Compared with GLUT1, GLUT3 has a significantly higher affinity for glucose.^38^ A study by Cheng et al revealed that the down-regulation of Ras related glycolysis inhibitor and calcium channel regulator (RRAD) is closely associated with the progression and metastasis of OSCC. The study suggests that the down-regulation of RRAD increases intracellular Ca^2+^ concentration, which subsequently activates the calcium/calmodulin-dependent protein kinase IV (CAMKIV)-cAMP responsive element binding protein 1 (CREB1) axis, ultimately leading to the up-regulation of GLUT3.^39^ Cancer cells often utilize fructose as an alternative energy and carbon source to drive glycolysis and support the synthesis of various biomolecules. GLUT5 is the only fructose-specific transporter. A study by Huang et al demonstrated that the IL-6/signal transducer and activator of transcription 3 (STAT3) axis promoted GLUT5 expression, facilitating fructose uptake and utilization, thereby enhancing OSCC cell growth.^40^ These complex molecular interactions highlight the significant influence of glucose metabolism regulation on the metastatic potential of OSCC cells.

The influx of glucose into the cellular cytoplasm initiates a cascade of chemical transformations, during which hexokinase 2 (HK2) serves as the initial rate-limiting enzyme.^41^ circMDM2 plays a pivotal role in augmenting HK2 activity by selectively targeting miR-532-3p, thereby promoting glycolysis in the context of OSCC.^42^ On another front, bergenin, a naturally occurring compound, has been identified as a significant inhibitor of OSCC cell growth. This effect is attributed to its inhibitory impact on HK2 expression and AKT phosphorylation.^41^ Tanshinone IIA (Tan IIA), derived from Danshen, emerges as a compelling compound with the capacity to impede HK2-mediated glycolysis. Tan IIA achieves this via influencing the stability of c-Myc and inhibiting AKT phosphorylation, ultimately curtailing HK2 expression within OSCC cells.^43^ Gastrodin, a naturally occurring compound, may serve as a potential therapeutic candidate for OSCC. A study suggests that gastrodin inhibits glucose metabolism by suppressing HK2 expression via AKT signaling, thereby reducing cisplatin resistance.^44^ AKT, also known as protein kinase B, is a crucial cell-signaling molecule. Its activation is involved in various biological processes, including cell growth, migration, and metabolism.^45^ The abnormal activation of AKT is closely linked to the development of numerous tumors. TRIM31 is a protein that belongs to the TRIM family. It is involved in regulating cellular behavior, gene transcription,^46^ and protein ubiquitination.^47^ In OSCC, TRIM31, a promoter of glycolysis, facilitates AKT phosphorylation and promotes cancer cell proliferation.^48^ Clinically, down-regulating AKT expression is expected to be a means to enhance the prognosis of OSCC patients. Furthermore, the phosphoinositide-dependent kinase 1 (PDK1) inhibitor BX795 has emerged as a new therapeutic pathway with the potential to reshape the glycolytic landscape in OSCC. BX795 suppresses glycolysis through down-regulating the PDK1/cluster of differentiation 47 (CD47)/AKT-mediated glycolytic signaling pathway, thereby increasing the sensitivity of OSCC patients to radiotherapy and improving treatment outcomes.^49^ The link between circadian rhythm disruption and cancer pathogenesis has become a key area of research. For instance, the circadian gene TIMELESS is highly expressed in patients with OSCC and is associated with poor prognosis. Further studies suggest that TIMELESS regulates glucose metabolism by promoting the expression of GLUT1, HK2, and pyruvate kinase M2 (PKM2), thereby facilitating oral cancer cell proliferation.^50^

Glucose can be converted to lactate in the cytoplasm, thereby impacting the cytoplasmic pH level. Monocarboxylate transporters (MCTs) are passive membrane transport proteins that are essential for the maintenance of intracellular pH.^51^ This is because when the internal level of lactate exceeds the external, lactate flows out of the cell, thus maintaining the normal pH of the cell.^52^ Interestingly, cancer cells often prefer glycolysis for glucose metabolism, leading to a significant accumulation of lactic acid in the cell, which hinders the rapid proliferation of cancer cells.^51^ Consequently, MCTs facilitate lactate excretion from the cell to ensure that normal intracellular pH levels are maintained. Furthermore, excessive extracellular lactate results in immune resistance in HNSCC, as the abundance of lactate suppresses the proliferation and function of monocytes and cytotoxic T cells.^52^ Hence, MCTs play a crucial role in the advancement of HNSCC. Notably, the expression of MCTs is elevated in various cancers, including HNSCC, especially in OSCC.^53^^,^^54^ Moreover, the expression of MCT4 is closely associated with the staging and classification of OSCC, which adversely affects the prognosis for patients.^55^

HNSCC cell lines show a preference for lactate metabolism compared with glucose metabolism.^56^ Elevated expression of MCT1 and MCT4 in HNSCC cell lines enhances lactate uptake.^57^ This metabolic preference saves numerous intermediate steps and energy compared with oxidative glucose metabolism, producing more ATP per molecule of lactate than per molecule of glucose.^56^ When excessive lactate in glycolysis-prone HNSCC cell lines is excreted through MCT passive membrane transport, that contrasts with those favoring oxidative lactate metabolism, which use MCT transport proteins to import external lactate, thereby converting it to pyruvate for the tricarboxylic acid cycle.^56^ Therefore, targeting MCT represents a potential strategy for controlling tumor growth. Subsequently, in the presence of lactate dehydrogenase isoenzyme 1 (LDH-1), lactate is converted into pyruvate, while NAD^+^ is converted to NADH and H^+^, providing substrates for the tricarboxylic acid cycle.^58^ This process demonstrates how cells adapt to hypoxic conditions by modifying metabolic pathways. Lactate and the produced pyruvate are the primary promoters of angiogenesis. Interestingly, pyruvate can act on hypoxia-inducible factor 1-alpha (HIF-1α) and prolyl hydroxylases (PHD).^59^ In the presence of oxygen and cofactors such as vitamin C, PHD can target HIF-1, which participates in and catalyzes the hydroxylation of HIF-1 residues.^60^ Activation of HIF-1 promotes angiogenesis by promoting the transcription of vascular endothelial growth factor A (VEGFA) and VEGFR2.^60^ Similarly, lactate promotes the activation of IκB kinase β (IKKβ), leading to the transcription of interleukin 61. Moreover, lactate and pyruvate can regulate the expression of hypoxia-related genes, thereby promoting the expression of MCT1, MCT2, and MCT4, indicating that lactate plays a positive feedback role in the regulation of glycolysis.^62^^,^^63^ In addition, angiogenic factors such as VEGF and hyaluronic acid mediate the up-regulation of exogenous lactate, participating in the invasion and metastasis of HNSCC.^64^ Therefore, targeting MCT is a potential measure to control tumor growth.

**Figure 1:**
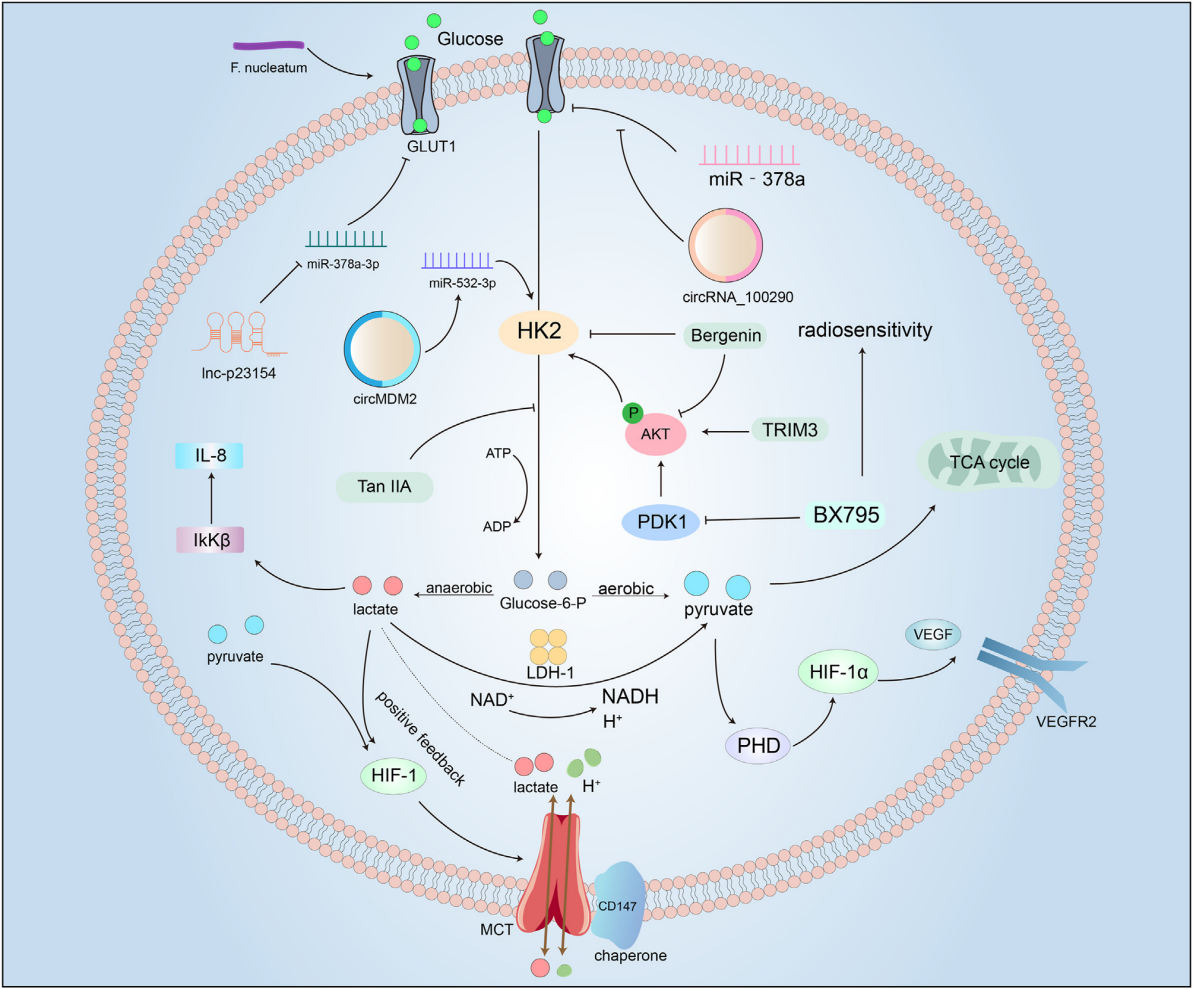
Roles of glucose metabolism in head and neck cancer. Glucose enters the cell via glucose transporter 1 (GLUT1), a process regulated by several non-coding RNAs. lnc-p23154 enhances GLUT1 expression by repressing miR-378a-3p transcription. circRNA_100290 acts as a ceRNA of miR-378, counteracting the inhibitory effects of miR-378a on GLUT1, promoting glycolysis. Glucose is converted to glucose-6-phosphate, which is catalyzed by the key enzyme hexokinase 2 (HK2). circMDM2 enhances HK2 activity by targeting miR-532-3p. Compounds such as Tan IIA and bergenin inhibit glycolysis by suppressing HK2. Tripartite motif-containing 31 (TRIM31) and phosphoinositide-dependent kinase 1 (PDK1) promote HK2 expression by enhancing protein kinase B (AKT) phosphorylation, while the PDK1 inhibitor BX795 inhibits PDK1 expression. Subsequently, glucose-6-phosphate can be converted to pyruvate or lactate under aerobic or anaerobic conditions. Lactate can be converted back to pyruvate by lactate dehydrogenase isoenzyme 1 (LDH-1). Pyruvate targets prolyl hydroxylases (PHD) and hypoxia-inducible factor 1 (HIF-1), with HIF-1 promoting monocarboxylate transporter (MCT)-mediated lactate transport. Lactate entering the cell can provide positive feedback by targeting HIF-1. PHD activates HIF-1, which in turn promotes vascular endothelial growth factor (VEGF) expression.

CD147 is a transmembrane glycoprotein, also known as Basigin or EMMPRIN, that is part of the immunoglobulin superfamily.^65^ CD147 is upregulated in several cancers, including HNSCC, and can promote the progression of HNSCC through activating the nuclear factor-kappa B (NF-κB) signaling pathway.^66^ It plays a critical role as a chaperone protein, contributing to the correct spatial conformation of MCT1 and MCT4, thus enhancing their function, stability, and expression.^65^^,^^67^ Significantly, it has been shown that CD147 is crucial in MCT-mediated lactate transport, maintaining a persistent association with MCTs on the plasma membrane.^68^ Histone lactylation, a recently discovered epigenetic modification, plays a crucial role in linking lactate accumulation to tumor immune evasion. Studies have shown that lactate promotes METTL3 up-regulation through H3K18 lactylation, enhancing RNA m6A modification and contributing to immune suppression in colorectal cancer. Additionally, histone lactylation regulates M1/M2 macrophage polarization, further promoting immune evasion. In head and neck cancer, lactate induces H3K9 lactylation and enriches the IL-11 gene region. IL-11, via the Janus kinase 2 (JAK2)/STAT3 signaling pathway, causes CD8^+^ T cell dysfunction. Notably, IL-11 knockout restores CD8^+^ T cell function and significantly enhances the efficacy of immunotherapy.^69^ These molecular insights open the way for precision interventions targeting key players in the complex network of glycolytic regulation, facilitating novel strategies for the management of HNSCC (Fig. 1).

## Roles of lipid metabolism in head and neck cancer

Lipids consist of fatty acids and several other substances, such as cholesterol and phospholipids. They are synthesized by the endoplasmic reticulum and contribute to the composition of many substances in the body. For instance, phospholipids, a type of lipid, are the main components of cell membranes and organelles. Meanwhile, lipids perform various functions within the body in addition to their structural contributions. For example, they serve as the primary form of energy storage, provide energy to the organism, and participate in the transmission of information, among other roles.^70^ This multifaceted function makes lipids indispensable in life processes. Cancer development is closely associated with lipid metabolism, especially during the initial stages of cancer development when lipid uptake and synthesis are increased.^71^ This emphasizes the intricate interactions between lipid dynamics and the development of tumors.

Obesity results from a metabolic imbalance, leading to an overaccumulation of adipose tissue in the body.^72^ In a complex microenvironment, cancer cells are influenced by numerous cellular and chemical factors, of which adipocytes and obesity-related inflammation (*e.g.*, white adipose tissue inflammation) are particularly significant in facilitating the development of the tumor microenvironment.^73^^,^^74^ Furthermore, obesity is recognized as an independent risk factor for OSCC in the clinic. It has been observed that patients with obesity-related OSCC typically exhibit a worse prognosis than those who are not obese.^75^ CD36 is a transmembrane glycoprotein that acts as a receptor and transporter for fatty acids, facilitating the uptake of exogenous fatty acids into cells. Studies have shown that CD36 is highly expressed in various tumor types, including HNSCC, with its expression in OSCC being 40 times higher than that in normal tissues.^76^, ^77^, ^78^, ^79^ Dietary palmitic acid, a saturated fatty acid, enhances CD36 activity by increasing the supply of fatty acids, thereby promoting the growth of OSCC.^80^ This suggests that dietary regulation of CD36 could influence the progression of OSCC.

Acetyl-CoA, the key initiator of fatty acid synthesis, is generated in the cytoplasm by ATP-citrate lyase (ACLY) through the cleavage of citrate. The up-regulation of ACLY in cancer cells is associated with poor prognosis in patients with HNSCC.^81^ In nasopharyngeal carcinoma, lncRNA TINCR promotes cancer cell proliferation, metastasis, and chemotherapy resistance by inhibiting ACLY ubiquitination degradation and regulating acetyl-CoA metabolism.^82^ Acetyl-CoA carboxylase (ACC), a rate-limiting enzyme in fatty acid synthesis, catalyzes the conversion of acetyl-CoA to malonyl-CoA and is regulated by AMP-activated protein kinase (AMPK). Mutations in ACC and the loss of AMPK phosphorylation lead to resistance of HNSCC cells to cetuximab. ACC inhibitors can mitigate cetuximab resistance in HNSCC.^83^ Fatty acid synthase (FASN) is a key enzyme in fatty acid synthesis, catalyzing the conversion of acetyl-CoA and malonyl-CoA into long-chain fatty acids. The up-regulation of FASN in HNSCC is associated with poor prognosis and lung metastasis.^84^^,^^85^ Orlistat, an inhibitor of FASN, shows promising effects in inhibiting OSCC proliferation and metastasis.^86^ Carboxylesterase 2 (CES2) is a lipase that can inhibit the *de novo* synthesis of fats, induce the hydrolysis of triglycerides, and regulate lipid metabolism. In addition, CES2 regulates glucose metabolism, thereby enhancing insulin secretion and sensitivity, reducing blood glucose levels, and increasing glucose tolerance.^87^ Furthermore, CES2 specifically regulates lipid metabolism in OSCC by hydrolyzing diglycerides and inhibiting the formation of membrane phospholipids in OSCC cells, which significantly reduced the proliferation of OSCC cells. Additionally, CES2 inhibited the metastatic ability of OSCC cells by suppressing the activation of the PI3K/AKT/MYC oncogenic signaling pathway and hindering the process of epithelial–mesenchymal transition in OSCC cells.^88^ In recent years, the important role of non-coding RNAs in lipid metabolism has gained increasing attention. Studies have shown that miR-122-5p regulates hepatic lipid metabolism by inhibiting the translation of cholesterol 7α-hydroxylase.^89^ The miR-31-5p-acyloxyacyl hydrolase 1 (ACOX1) (a rate-limiting enzyme in peroxisomal β-oxidation) axis promotes OSCC progression by modulating cellular lipid metabolism. Further investigation revealed that prostaglandin E2, a key substrate for ACOX1-mediated peroxisomal β-oxidation, enhances tumor cell invasiveness by activating the extracellular signal-regulated kinase (ERK)-matrix metallopeptidase 9 (MMP9) signaling pathway.^90^

**Figure 2:**
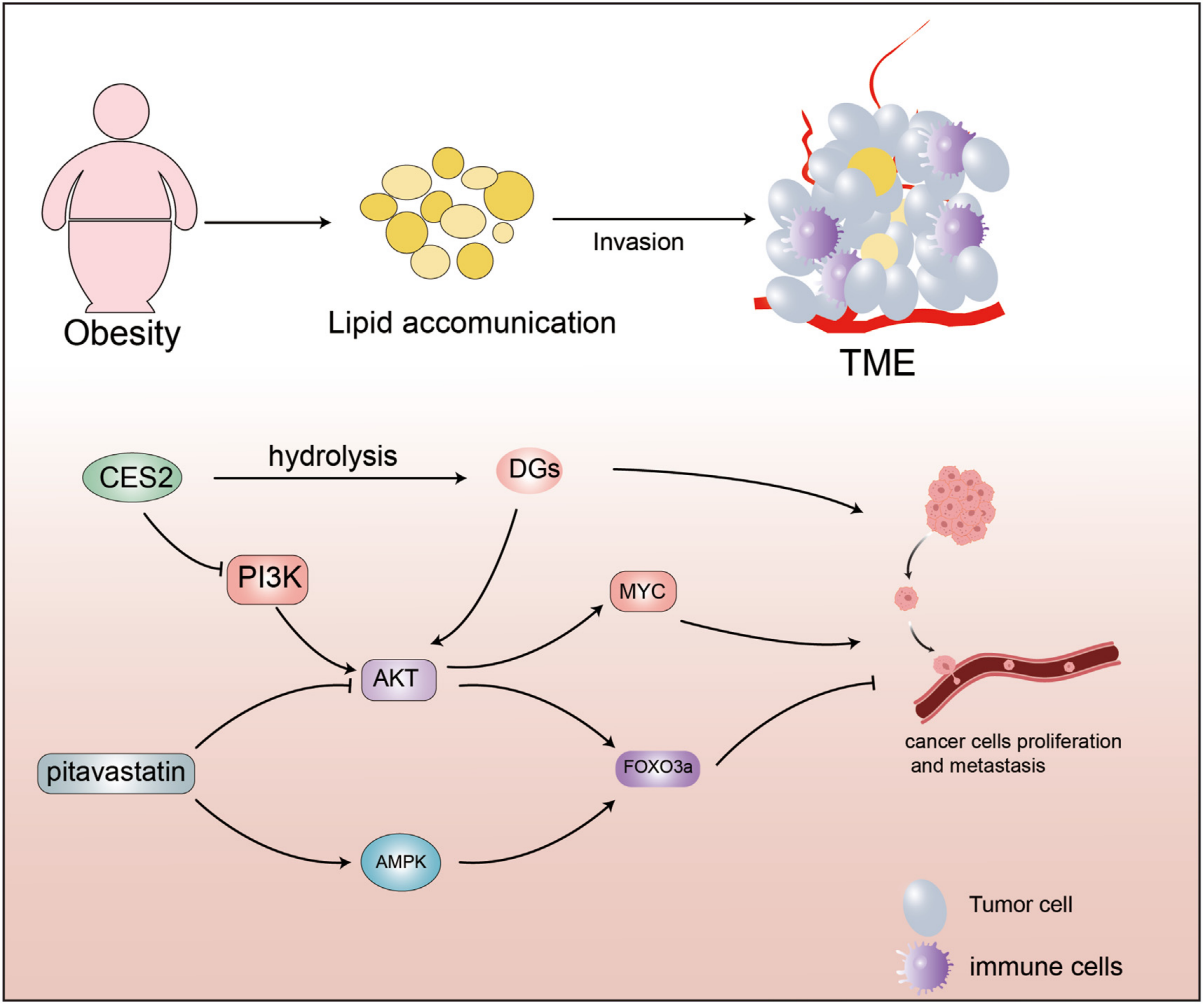
Roles of lipid metabolism in head and neck cancer. The accumulation of adipose tissue due to obesity enhances the tumor microenvironment, thereby promoting tumor progression. Carboxylesterase 2 (CES2) plays a crucial role in lipid metabolism by inhibiting the *de novo* synthesis of fats. CES2 suppresses the activation of the phosphoinositide 3-kinase (PI3K)/protein kinase B (AKT)/MYC oncogenic signaling pathway, thereby hindering tumor progression. Diglycerides (DGs) promote the progression of oral squamous cell carcinoma (OSCC) by enhancing the formation of phospholipids in cell membranes and the AKT/MYC pathway. CES2 inhibits the development and progression of OSCC by hydrolyzing DGs. Pitavastatin promotes the nuclear translocation of forkhead box class O 3a (FOXO3a) via AKT and AMP-activated protein kinase (AMPK), thereby inhibiting the metastasis of OSCC.

Abnormal cholesterol metabolism is closely associated with phenomena such as chemotherapy resistance and immune evasion in tumors.^91^ Sterol regulatory element-binding proteins (SREBPs), a family of transcription factors located in the endoplasmic reticulum, are primarily involved in regulating cholesterol metabolism. SREBPs are encoded by SREBP1 and SREBP2. SREBP1 acts as a key transcription factor in fatty acid synthesis and promotes immune cell infiltration in HNSCC by up-regulating steroidogenic acute regulatory protein-related lipid transport protein 4 (STARD4).^92^, ^93^, ^94^, ^95^ Furthermore, the natural compound resveratrol regulates SREBP1 expression, inhibits lipid metabolism, and induces autophagy in OSCC. SREBP2 plays a central role in the regulation of cholesterol synthesis.^96^ Anterior gradient protein 2 (AGR2), an endoplasmic reticulum-resident protein, enhances SREBP2-mediated cholesterol metabolism and cisplatin resistance by activating the AKT signaling pathway.^97^ The natural compound quercetin down-regulates AGR2 in a concentration-dependent manner, inhibiting cholesterol metabolism and cisplatin resistance in OSCC.^98^ Statins are a class of drugs used in the treatment of high cholesterol and hyperlipidemia that work by inhibiting HMG-CoA reductase, a key enzyme involved in cholesterol synthesis, to reduce blood lipid levels. A recent study showed that pitavastatin not only induced apoptosis in OSCC cells SCC15 but also inhibited the metastasis of these cells.^99^ Forkhead box class O (FOXO) subfamily member FOXO3a is a transcription factor that plays a significant role in regulating cell proliferation and apoptosis,^100^ thereby maintaining cellular homeostasis, adapting to environmental changes, and regulating life processes. Importantly, pitavastatin inhibits the AKT/FOXO3a pathway and promotes the AMPK/FOXO3a pathway, leading to the nuclear translocation of FOXO3a and effectively inhibiting the metastasis of OSCC cells.^101^^,^^102^ Lipid metabolism plays a crucial role in tumor cell growth and immune evasion. Understanding its key functions in the tumor microenvironment offers new insights for the development of targeted therapeutic strategies. (Fig. 2).

## Amino acid metabolism in head and neck cancer

Amino acid metabolism plays a crucial role in tumorigenesis, and various tumor types exhibit specific amino acid dependencies.^103^ To better understand this phenomenon, it is important to recognize how amino acids in the human body have traditionally been classified into essential and non-essential amino acids based on nutritional requirements.^104^ Essential amino acids are those that the body cannot synthesize on its own and thus must be obtained from food.^105^ In the stomach and small intestine, exogenous proteins are broken down into amino acids and oligopeptides by various proteases, which are then absorbed through specific amino acid transporters and the glutamine–glutamate cycle.^106^ Conversely, non-essential amino acids are those that the body can synthesize on its own. In the body, amino acids are not only synthesized in the form of proteins but are also involved in a number of important biological processes. Adequate consumption of amino acids is vital for human growth, development, and the maintenance of vital activities. In cancer, the extensive amino acid requirements of cancer cells can be met by reprogramming amino acid metabolism to increase the intake of essential amino acids from foods.^107^^,^^108^ Interestingly, tumor growth is constrained when the body is deficient in essential amino acids. Further research has revealed that amino acid metabolism can modulate various immune cells.^109^ This demonstrates that amino acid metabolism is closely related to tumor proliferation, metastasis, and immune tolerance.

Hans Krebs initially characterized the synthesis of glutamine, introduced the Krebs cycle, and noted the significance of glutamine in maintaining biological homeostasis.^110^ Despite a long history of research on the role of glutamine, studies on glutamine in the immune system have only recently begun. Glutamine, as the second primary nutrient for tumor cells, supplies both nitrogen and carbon sources to the organism, thereby significantly impacting tumor development.^111^^,^^112^ This is especially critical because tumor cells require glutamine more than other amino acids,^113^ and strategies such as limiting amino acids or inducing starvation can disrupt tumor metabolism, thus inhibiting tumor cell growth.^114^ Studies utilizing tracer experiments have determined that proteins supplied by glutamine account for more than 50% of the exogenous proteins in cancer cells.^115^^,^^116^

Transcellular membrane transport of glutamine requires the involvement of alanine-serine-cysteine transporter 2 (ASCT2), a crucial Na^+^-dependent amino acid transporter protein located mainly in the cell membrane and encoded by the SLC1A5 gene, which is essential for transporting glutamine and other amino acids.^117^ Remarkably, the expression of ASCT2 was significantly elevated in OSCC compared with normal cells and adversely affected the overall survival rate of OSCC. Thus, the knockdown of the ASCT2 gene not only efficiently decreases glutamine uptake but also suppresses glutathione synthesis, increases reactive oxygen species levels, and ultimately leads to OSCC cell apoptosis.^118^ Furthermore, glutamine facilitates the transport of other essential amino acids and activates the phosphorylated mammalian target of rapamycin (p-mTOR)/phosphorylated S6 ribosomal protein (p-S6) signaling pathway, which promotes cell growth and proliferation.^118^, ^119^, ^120^

Moreover, the entry of glutamine into mitochondria and its involvement in amino acid metabolism within mitochondria is crucial for effector T cells that require substantial amounts of ATP.^121^ Furthermore, glutathione is a tripeptide composed of glutamate, cysteine, and glycine, with antioxidant functions. The catabolism of glutamine can promote the synthesis of glutathione, which regulates the activity of T cells.^122^ Furthermore, mTOR is involved in the regulation of this process^122^^,^^123^ and plays important roles in the metabolic activities of different T cells exhibiting various metabolic preferences. mTOR complex 1 (mTORC1) and mTORC2 are both able to modulate the differentiation and function of T cells via distinct signaling pathways. Interestingly, inhibition of mTOR activity is known to favor the generation of forkhead box P3 (FOXP3)-positive regulatory T cells (Tregs) while simultaneously hindering the formation of effector T cells.^124^, ^125^, ^126^ It has been shown that the down-regulation of glutamine, leucine, and glucose transporters through gene editing reduces mTOR activity, thereby inhibiting the generation of effector cells.^127^, ^128^, ^129^ Consistent with these mechanisms, it has been shown that glutamine restriction through nutritional means can stimulate the T cell receptor, leading to the differentiation of naive CD4^+^ T cells into Tregs.^130^

Glutamine synthetase facilitates the transformation of glutamate and ammonia into glutamine and water. During radiotherapy, nasopharyngeal carcinoma cells undergo cell death. However, the expression of glutamine synthetase is increased in some cancer cells in response to radiation stimulation. This adaptation allows the survival of certain cells and may lead to resistance to radiotherapy. This resistance could be due in part to the role of glutamine synthetase in regulating the cell cycle, which promotes the restoration of G2/M stage cancer cells.^131^ Moreover, in nasopharyngeal carcinoma, glutamate dehydrogenase is overexpressed, which is further exacerbated by catalyzing the transformation of glutamine into α-ketoglutarate.^115^ This metabolic alteration allows elevated α-ketoglutarate in nasopharyngeal carcinoma cells to act as a substrate for the tricarboxylic acid cycle, which continually provides energy support for cancer growth.

Studies have shown that arginine, as a semi-non-essential amino acid, plays a role in the formation of proteins, urea, nitric oxide, and creatine.^132^ Despite the body's ability to synthesize arginine, external sources are usually required to meet the body's needs. For this reason, cationic amino acid transporters (CAT), encoded by solute carrier family 7 member A (SLC7A), play a crucial role in regulating arginine uptake at the cell membrane.^133^^,^^134^ Within the cellular signaling landscape, Ras-related GTP binding protein (Rag GTPase), a small GTP-binding protein of the Ras superfamily, and solute carrier family 38 member 9 (SLC38A9), a crucial transmembrane protein found on lysosomes, work in concert. SLC38A9 interacts with Rag GTPase to detect fluctuations in arginine levels, thus regulating mTORC1 activity. Moreover, SLC38A9 acts as a scaffolding protein, anchoring Rag GTPase and mTORC1 to the lysosome surface.^135^^,^^136^ These interactions enable arginine to modulate the activity of SLC38A9, resulting in changes in the distribution of arginine in the cytoplasm and lysosomes and affecting the activity of mTORC1. Activation of mTORC1 occurs when SLC38A9 signals arginine enrichment.^136^^,^^137^ Notably, due to the low affinity of SLC38A9 for arginine, it usually functions at elevated arginine concentrations.^138^ The cytoplasmic arginine sensor for mTORC1 subunit 1 (CASTOR1) is a cytoplasmic protein that acts as an arginine sensor but has a higher affinity for the amino acid.^139^ When arginine is abundant, CASTOR1 binds to arginine. On the other hand, under arginine-deficient conditions, CASTOR1 binds to gap activity toward Rags 1 (GATOR2), weakening the inhibitory effect of GATOR2 on GATOR1. This interaction results in the dissociation of the GATOR1/GATOR2 complex (a regulator upstream of mTORC1) and subsequently reduces mTORC1 activity.^139^^,^^140^ With the maintenance of homeostasis between SLC38A9 and CASTOR1 *in vivo*, mTORC1 activity is kept within an appropriate range to prevent overactivation. This regulation is crucial for the proliferation and differentiation of both tumor cells and immune cells.^109^ Moreover, arginine regulates the cell cycle. Specifically, arginine deficiency enhances mTORC2 activity and reduces mTORC1 activity, leading to cell cycle arrest.^141^ Interestingly, this cell cycle arrest caused by arginine deprivation can be reversed by arginine supplementation.^141^ Additionally, the overexpression of SLC38A9 *in vivo* can restore the decrease in mTORC1 activity caused by arginine deficiency.^136^^,^^137^ Interestingly, T cell proliferation and activity decrease with arginine reduction, but arginine supplementation restores the activity of T cells.^142^, ^143^, ^144^, ^145^

**Figure 3:**
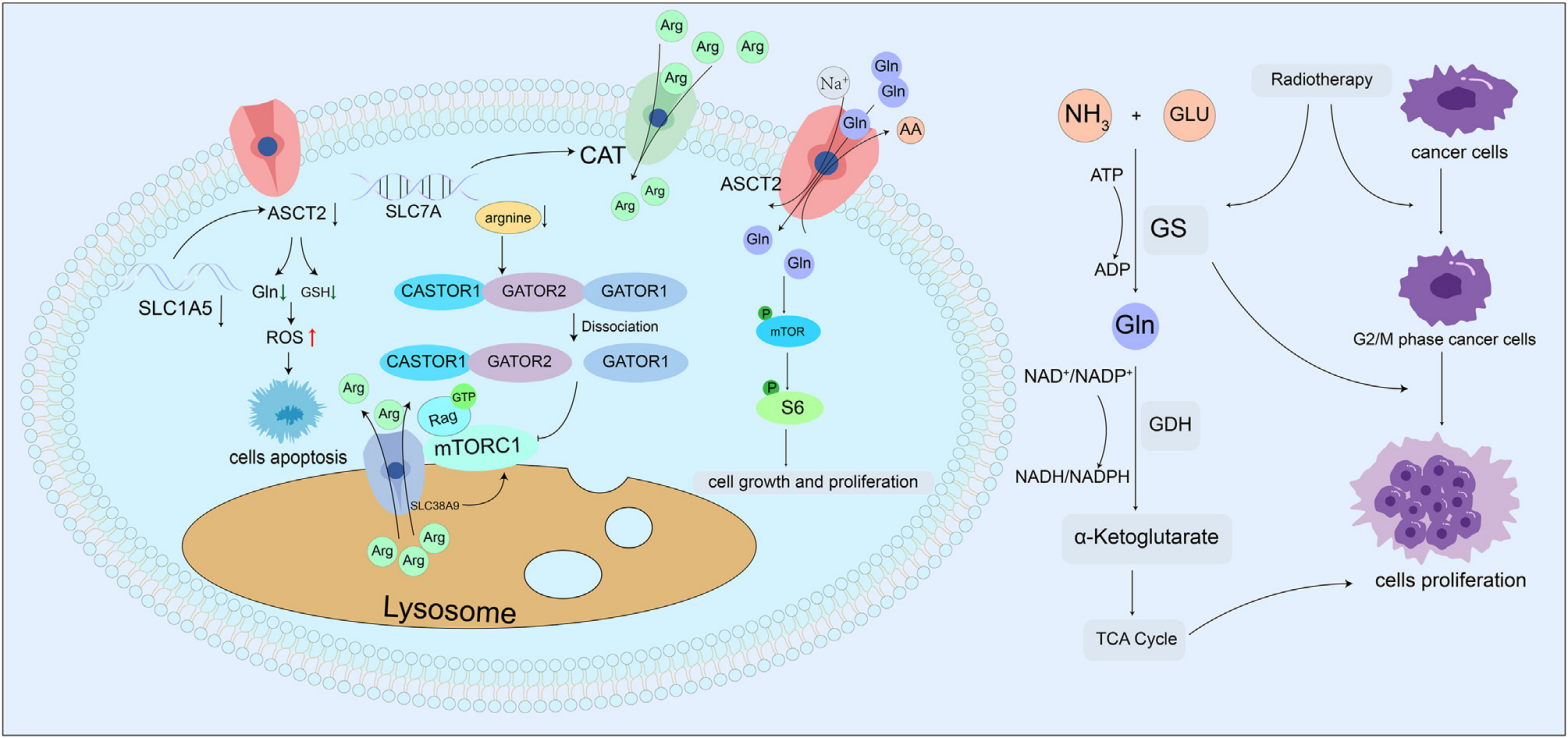
Amino acid metabolism in head and neck cancer. Regulation of amino acid metabolism in head and neck squamous cell carcinoma (HNSCC) and the mechanisms of HNSCC radioresistance. Arginine enters the cell via cationic amino acid transporters (CAT), encoded by solute carrier family 7 member A (SLC7A). When intracellular arginine levels decrease, the cytoplasmic arginine sensor for mTORC1 subunit 1 (CASTOR1) binds to gap activity toward Rags 2 (GATOR2), leading to the dissociation of the GATOR1/GATOR2 complex and subsequently reducing mTORC1 activity. When lysosomal arginine levels increase, the enhanced activity of solute carrier family 38 member 9 (SLC38A9) not only alters the distribution of arginine between the lysosome and cytoplasm but also increases mTORC1 activity. Alanine-serine-cysteine transporter 2 (ASCT2), encoded by solute carrier family 1 member 5 (SLC1A5), is a Na^+^-dependent alanine-serine-cysteine transporter, with glutamine being its primary substrate. Glutamine activates the p-mTOR/p-S6 signaling pathway, which promotes cell growth and proliferation. Reduced glutamine uptake promotes reactive oxygen species (ROS) production, leading to cell apoptosis. On one hand, radiation therapy causes cancer cells to accumulate in the G2/M phase. On the other hand, radiation therapy enhances the activity of glutamine synthetase, which not only promotes the restoration of G2/M phase cancer cells but also promotes cell proliferation by enhancing the tricarboxylic acid cycle.

Arginine biosynthesis requires the involvement of argininosuccinate synthetase and argininosuccinate lyase.^132^^,^^146^ Argininosuccinate synthetase 1 (ASS1) plays an important role in the urea cycle by catalyzing the formation of argininosuccinate from arginine and fumarate and is a key enzyme in inhibiting the metabolism of nasopharyngeal carcinoma.^115^ Through metabolic transformation, arginine is converted into urea and l-ornithine by arginase-1 (Arg1),^133^ which, under the action of nitric oxide synthase, produces l-citrulline and nitric oxide, which acts as a signaling molecule in the body and is involved in the transmission of neurotransmitters to regulate the activity of immune cells.^147^ Ultimately, arginine deiminase can break down arginine into citrulline.^148^ It was found that if the growth environment of HNSCC cells was deficient in arginine, it reduced the proliferation capacity of these cancer cells, and arginine deiminase-based arginine deprivation therapy inhibited all cell lines of HNSCC.^149^ This insight provides a potential therapeutic approach for the treatment of HNSCC (Fig. 3).

## Immune dysregulation in head and neck cancer

### Dysfunction of the antigen presentation pathway in head and neck cancer

The transformation from a normal cell to a cancer cell involves the accumulation of a series of events that disrupt standard cellular control mechanisms.^150^ During this process, cancer cells undergo differentiation, which is characterized by significant alterations in their molecular profiles, gradually differentiating them from normal cells. A representative example is the major histocompatibility complex class I (MHC-I) on cancer cell surfaces, which binds to neo-differentiation antigens triggered by these events. The MHC-I is a protein molecule that specifically binds to intracellular antigenic fragments and displays them on the cell surface so that they are recognized by CD8^+^ T cells, thus eliciting a T cell reaction.^151^ Furthermore, calnexin, calreticulin, endoplasmic reticulum protein 57 (ERp57), and tapasin are chaperone proteins for MHC-I, which work together to enhance the binding of MHC-I to β2-microglobulin and to facilitate its interaction with immunogenic peptides, thereby effectively presenting it extracellularly.

To eliminate tumors, the immune system must deal with a series of events. Immunogenic peptides must be transported to the endoplasmic reticulum lumen in the presence of the transporter associated with antigen processing 1 (TAP1) and processed into optimal-sized fragments by endoplasmic reticulum aminopeptidase 1 (ERAP1), which then bind to MHC-I.^152^ After being processed, the MHC–I complex is moved to the Golgi apparatus and then transported to the cell membrane via vesicles.^153^ Antigens present on MHC-I are captured by dendritic cells and presented to T cells, leading to the activation of T cells. This activation prompts the activated effector T cells to migrate to the tumor tissue, inducing its death. Furthermore, antigens released from the dead tumor tissue further engage the immune system in a broader and deeper immune cycle against cancer. This response helps to recognize and eliminate additional potential cancer cells, thus enhancing the immune defense against cancer.^154^ However, tumor cells can evade immunosuppression through multiple mechanisms. For example, HNSCC cells express PD-L1 on their surfaces to facilitate immune escape, and PD-L1 interacts with programmed cell death protein 1 (PD-1) on the surface of T cells to suppress its activity, thereby escaping immunosuppression.^155^ PD-L1 is expressed in multiple tumors, including HNSCC.^156^ The transcription factor Snail and the cytoskeletal protein vimentin play key roles in the epithelial–mesenchymal transition process of cells. It has been demonstrated that PD-L1 can promote the epithelial–mesenchymal transition of HNSCC cells via the glycogen synthase kinase 3 beta (GSK3β)/Snail/vimentin axis.^157^ Furthermore, the study showed that the methanol extract of I. okamurae (MEIO) can suppress the PD-L1/Snail/vimentin axis, which can be used as a potential anti-cancer drug for HNSCC. lncRNAs also play a vital role in promoting immune escape by regulating various immune processes in the tumor microenvironment.^158^ Shi et al identified interferon-induced transmembrane protein 4 pseudogene (IFITM4P) as an immune-associated lncRNA with upregulated expression in OSCC. Bioinformatics analysis showed that IFITM4P could induce PD-L1 expression, thus promoting immune escape. Importantly, PD-L1 blocker (PD-1 mAb) showed significant anti-cancer impact on tumor-bearing mice with elevated IFITM4P expression.^159^ Additionally, cancer-associated fibroblasts (CAFs) play an important role in the progression of OSCC cells by promoting tumor growth and modulating the immune response.^160^, ^161^, ^162^, ^163^ In particular, CAFs enhance the progression of OSCC by secreting cytokine IL-33, which binds to its receptor suppression of tumorigenicity 2 (ST2), thereby enhancing PD-L1 synthesis and expression.^164^^,^^165^

HNSCC cells may also evade immune responses by impacting MHC-I antigen presentation. Patients with higher MHC-I expression in HNSCC have longer disease-free survival than those with lower MHC-I expression.^166^ There are a number of reasons for this difference, and one potential reason is related to the antigen presentation mechanism (APM). This is particularly important because the CD8^+^ T cells play a key role in adaptive immunity. Specifically, the initial signal for CD8^+^ T cell activation is the presentation of MHC-I antigens. However, due to the reduced expression of transporters associated with TAP1, which is associated with antigen processing in HNSCC cells, cytotoxic T cells may be resistant to pathogens or cancer cells because they lack the essential signals to identify and eliminate these abnormal cells.^166^, ^167^, ^168^ Consequently, when the integrity of the tumor MHC-I APM is compromised, it results in the accumulation of MHC-I in the cytoplasm awaiting degradation and reduces the amount of MHC-I transported to the cell membrane.^169^ Through multiple mechanisms, HNSCC can reduce MHC-I antigen presentation, diminish CD8^+^ T cell activity, decrease interferon-gamma (IFN-γ) secretion, and ultimately become resistant to immunotherapy.^170^^,^^171^

Furthermore, non-coding RNAs can influence the post-transcriptional expression of antigen presentation-related genes through multiple methods.^172^^,^^173^ For example, lncRNA LINC02195 has the capacity to alter MHC-I expression in HNSCC, thus impacting antigen presentation. Moreover, LINC02195 expression was positively correlated with the number of CD8^+^ T cells in tumors and the patient prognosis,^174^ making it a potential target for HNSCC treatment. Additionally, another study showed that miR-9 can enhance the expression of MHC-I mRNA and TAP1 mRNA in nasopharyngeal cancer.^175^

Likewise, other factors such as human papillomavirus (HPV) infection can influence post-translational antigen presentation associated with MHC-I.^176^ Compared with HPV HNSCC, HPV ^+^ HNSCC has a weaker APM and lower T cell activation and proliferation.^177^ Furthermore, researchers have confirmed that proteins encoded by HPV can suppress antigen presentation.^176^^,^^178^ In particular, MHC-I can be inhibited by HPV 16 E5, reducing patient sensitivity to anti-PD-L1 immunotherapy and leading to immune resistance.^176^ Studies have shown that HPV 16 E7 can directly suppress TAP1 and MHC-I, inhibiting the presentation and processing of TAP1-associated antigen presentation.^178^^,^^179^

Some signaling pathways can disrupt the signaling of MHC-I APM. IFN-γ is produced by activated T lymphocytes and natural killer cells and plays a crucial role in the immune system. IFN signaling enhances the expression of APM-related components, which regulates MHC-I-mediated antigen presentation.^153^ It has been shown that IFN-γ pathway-associated gene deletions can impair antigen presentation in melanomas of the head and neck region, ultimately leading to immune escape and radiotherapy resistance in HNSCC.^180^ Moreover, IFN-γ effectively activates and phosphorylates signal transducer and activator of transcription 1 (STAT1), which leads to its transcription and subsequent increased TAP1 expression in HNSCC, resulting in significant enhancement of immune responses.^181^ However, the sustained activation of the PI3K signaling pathway has been shown to suppress the IFN-γ-pSTAT1 pathway, which reduces MHC-I expression and subsequently promotes immune escape from HNSCC. In addition, sustained activation of IFN-γ signaling can result in immune suppression by activating other pathways. For example, IFN-γ can trigger the activation of the WNT/β-catenin signaling pathway in melanomas, which leads to the proliferation and stemness of tumor cells.^182^ Similarly, multiple tumors, including HNSCC, have a reduced T cell infiltration due to the activation of the WNT/β-catenin signaling pathway, ultimately leading to reduced surrounding immune responses.^183^^,^^184^ Furthermore, in HNSCC cells, the activation of downstream interferon alpha and beta receptor subunit 1 (IFNAR1) signaling promotes the release of oncogenic exosomes carrying immune-regulatory factors that modulate immune responses, mediate immune suppression, and contribute to the development of cancer.^185^^,^^186^ Moreover, over 90% of HNSCCs overexpress the epidermal growth factor receptor (EGFR).^187^ It can be activated by epidermal growth factor (EGF) and similar factors, thereby triggering the activation of various signaling molecules, including Src homology region 2 domain-containing phosphatase-2 (SHP-2). This activation promotes the dephosphorylation of pSTAT1 and leads to the suppression of the transcription of APM-related components.^171^ Notably, in HNSCC cells, SHP-2 expression is more prevalent than SHP-1.^188^

HNSCC typically exhibits high levels of CD8^+^ T cell infiltration at the tumor core, yet their response to immune checkpoint inhibitors remains poor, suggesting the presence of multiple immunosuppressive factors that hinder effective anti-tumor immune responses. For example, Tregs and CD163^+^ macrophages contribute to immune evasion by suppressing CD8^+^ T cell activation. In most HNSCC tumors, CD163^+^ macrophages and Tregs accumulate in large numbers at the tumor core and are closely associated with tumor progression. Further studies have shown that the spatial distribution of CD163^+^ macrophages and Tregs closely correlates with the proximity to CD8^+^ and CD4^+^ T cells, suggesting that interactions between these immune cells may regulate immune responses.^189^ Feng et al. found that the accumulation of Tregs within a 30 μm radius of CD8^+^ T cells correlated with improved patient prognosis.^190^ Meanwhile, Chiu et al suggested that oral OSCC induced macrophage differentiation into the M2 phenotype, thereby promoting tumor progression.^191^ Moreover, endoplasmic reticulum stress provides a pathway for tumor cells to evade immune suppression through various mechanisms.^192^, ^193^, ^194^ Under endoplasmic reticulum stress, exosomes released by OSCC cells induce the polarization of M2 macrophages, which play a pivotal role in facilitating cancer progression through mechanisms including angiogenesis, immunosuppression, tumor metastasis, and treatment resistance.^195^^,^^196^

### The interaction between energy metabolism and immune responses in head and neck cancer

Tumor cells reprogram their metabolism to meet their energy demands while simultaneously inhibiting immune responses through nutrient competition with immune cells.^23^ In tumor microenvironments, regions of high glucose metabolism are often associated with reduced immune cell infiltration or dysfunction, particularly in CD8^+^ T cells. This suggests that tumor cells may evade immune surveillance by “starving” immune cells through glucose metabolism.^197^ A study by Na et al demonstrated that the high metabolic state in HNSCC leads to alterations in the tumor microenvironment, resulting in T cell energy depletion and promoting tumor growth and expansion.^198^ Moreover, in a high-glucose metabolic environment, the proportion of M1 macrophages decreases, while that of M2 macrophages increases, thereby facilitating the establishment of an immunosuppressive microenvironment.^199^ Solute carrier family 2 member 3 (SLC2A3), a member of the SLC2A transporter family, belongs to the major GLUT family. In recent years, SLC2A3 has been highly expressed in various tumor types, including HNSCC, and its expression level is closely correlated with patient prognosis. Studies have shown that SLC2A3 plays a crucial role in glucose metabolism. In HNSCC, its expression is negatively correlated with the number of CD8^+^ T cells and resting dendritic cells, suggesting that SLC2A3 may promote tumor progression by inhibiting immune cell function.^200^ Key enzymes of glucose metabolism, PKM2 and phosphofructokinase (PFKP), are upregulated after radiotherapy, and metabolic reprogramming enhances tumor resistance to radiation. Inhibition of PKM2 and PFKP using Gannaitop significantly down-regulates the IL-8 signaling pathway and reduces radioresistance in HNSCC.^201^ SPP1^+^ macrophages promote tumor growth and metastasis. Studies indicate that SPP1^+^ macrophages have a high fructose metabolism profile and are more susceptible to metabolic intervention than other cells. Fructose inhibitors reduce the proportion of SPP1^+^ macrophages and increase the ratio of CD4^+^ to CD8^+^ T cells, thereby inhibiting HNSCC growth.^202^

Perilipin-3 (PLIN3), a surface protein of lipid droplets, plays a crucial role in regulating both lipid droplet biogenesis and degradation. Elevated expression of PLIN3 has been significantly associated with poor prognosis in patients with OSCC. Recent studies have demonstrated that PLIN3 promotes immune evasion in OSCC cells by inhibiting CD8^+^ T cell infiltration and up-regulating the expression of PD-L1 and B7–H2, which are ligands for PD-1.^203^ Sterol O-acyltransferase 1 (SOAT1) is a key enzyme in cholesterol metabolism, responsible for esterifying free cholesterol with fatty acids to form cholesteryl esters. This process is crucial for maintaining cellular cholesterol homeostasis and lipid balance. SOAT1 is highly expressed in OSCC and contributes to immune evasion by enhancing tumor cell lipid metabolism and inducing the polarization of M2 macrophages.^204^ Coiled-coil domain-containing 71 like (CCDC71L), a novel prognostic marker and potential immunotherapy target in the lipid metabolism of HNSCC, promotes tumor progression by enhancing the activity of Tregs and facilitating the polarization of M2 macrophages.^205^

**Figure 4:**
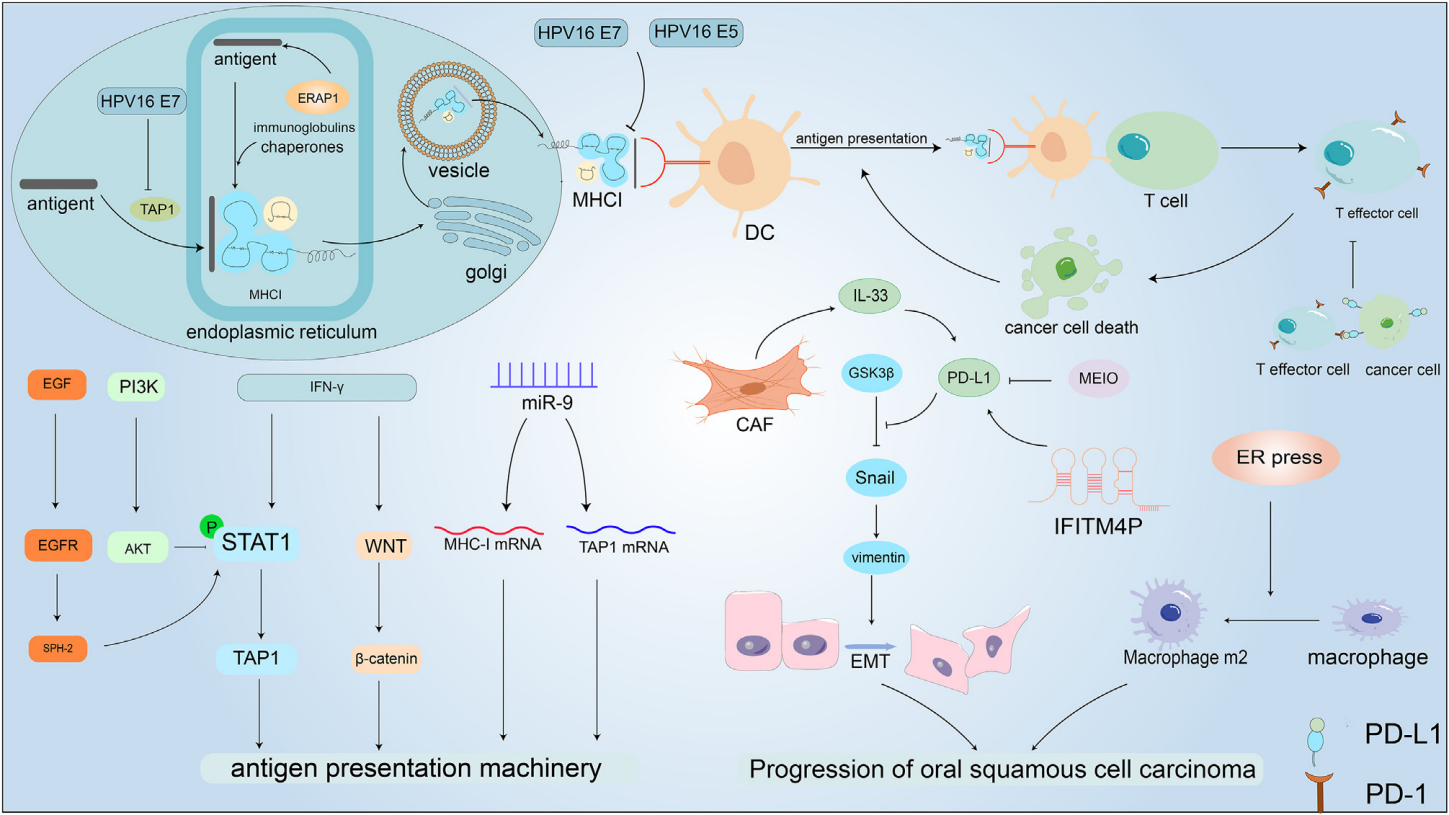
Immune dysregulation in head and neck cancer. Under the action of antigen processing 1 (TAP1), antigens are transferred to the endoplasmic reticulum (ER) lumen. After being trimmed to optimal size fragments by endoplasmic reticulum aminopeptidase 1 (ERAP1), they bind to MHC-I, then pass through the Golgi apparatus, and are finally transported to the cell membrane in vesicles, where they are presented on the cell surface. Dendritic cells recognize and present these antigens to T cells, leading to T cell activation. Activated T cells kill cancer cells, and the antigens released from the dying cancer cells further enhance antigen presentation. Antigen presentation is regulated by various mechanisms. miR-9 promotes the expression of MHC-I mRNA and TAP1 mRNA; interferon-gamma (IFN-γ) enhances antigen presentation by promoting TAP1 expression through increased signal transducer and activator of transcription 1 (STAT1) phosphorylation, which increases TAP1 expression in head and neck squamous cell carcinoma (HNSCC) and through the WNT/β-catenin axis. Cancer cells express programmed cell death protein 1 (PD-1) on their surface, which binds to programmed death-ligand 1 (PD-L1) on T cells, inhibiting T cell activity. PD-L1 promotes cancer progression by enhancing epithelial–mesenchymal transition (EMT) through the glycogen synthase kinase 3 beta (GSK3β)/Snail/vimentin pathway. Cancer-associated fibroblasts (CAFs) can promote PD-L1 expression by secreting interleukin-33 (IL-33); interferon-induced transmembrane protein 4 pseudogene (IFITM4P) also promotes PD-L1 expression, while the compound I. okamurae (MEIO) inhibits PD-L1 expression and shows promise as an anti-cancer drug for HNSCC. ER stress can induce the polarization of M2 macrophages, which promotes cancer progression.

Recent studies have highlighted the pivotal role of tryptophan metabolism in tumor immune evasion, inflammation, and autoimmune diseases. Tryptophan metabolites, including kynurenine and its derivatives, regulate T cell function and immune responses by activating specific receptors.^206^ High expression of indoleamine 2,3-dioxygenase in HNSCC catalyzes the conversion of tryptophan to l-kynurenine, thereby suppressing T cell proliferation and function.^207^, ^208^, ^209^, ^210^, ^211^ Further studies have shown that kynurenine up-regulates PD-L1 expression on granulocytic-myeloid-derived suppressor cells, macrophages, and dendritic cells and enhances PD-1 expression on CD8^+^ T cells in various malignancies. Additionally, kynurenine promotes the proliferation of Treg cells via activation of the aryl hydrocarbon receptor, thereby facilitating immune evasion in HNSCC.^206^^,^^212^ Glutamine also plays a critical role in shaping the tumor immune microenvironment. Its metabolic reprogramming influences the function of M2 macrophages, B cells, and natural killer cells and may promote immune suppression by down-regulating PD-1 expression on T cells.^213^ In HNSCC, high expression of SLC1A5 is associated with reduced CD8^+^ T cell infiltration, suggesting that SLC1A5 may suppress tumor immunity by modulating glutamine metabolism.^214^ Furthermore, combining cetuximab with glutamine uptake inhibitors has been shown to improve survival outcomes in HNSCC patients, likely by enhancing the immune responses and disrupting the tumor's metabolic adaptation.^215^ Understanding these intricate interactions offers valuable insights into the mechanisms of HNSCC progression and highlights potential avenues for targeted therapeutic interventions against this challenging malignancy (Fig. 4).

### Energy metabolism and immune response in head and neck cancer therapy

Currently, surgical resection, radiotherapy, and chemotherapy are the conventional treatments for HNSCC. Surgical resection aims to directly remove as much tumor tissue as possible, which is often most effective in the early stage of HNSCC. Conversely, radiotherapy and chemotherapy eradicate cancer cells using radiation and chemicals, respectively, while inhibiting tumor proliferation. Among the drugs used for HNSCC treatment, cetuximabhas emerged as the primary targeted therapy drug, which is particularly beneficial for patients with advanced HNSCC or those who are intolerant to traditional chemotherapy and can be used in combination with radiation therapy.^216^, ^217^, ^218^, ^219^ Cetuximab competitively blocks the binding of EGF to EGFR, thus inducing cell apoptosis and inhibiting the expression of molecules that play a significant role in tumor metastasis (*e.g.*, VEGF, MMP).^220^ Additionally, there exist several EGFR-targeted drugs that have yet to be approved, including the anti-EGFR monoclonal antibodies panitumumab and nimotuzumab, as well as erlotinib, afatinib, and dacomitinib, which work by inhibiting the EGFR tyrosine kinase.^221^

The reprogramming of energy metabolism in tumor cells and immune cells and their interactions facilitates the creation of a microenvironment for tumor cell growth and metastasis. The metabolites and immune effects produced by tumor cells and various cells in the tumor microenvironment are dynamically regulated. There are a series of drugs that have been developed by researchers that interfere with this process to varying degrees.

The malignant proliferation and invasive tendency of HNSCC can be effectively prevented by regulating glucose metabolism. This can be achieved through the regulation of glucose transporters and pivotal enzymes orchestrating glucose metabolism. The natural compounds bergenin and Tan IIA have been found to inhibit OSCC cell growth by inhibiting the activity of hexokinase and the phosphorylation of AKT.^41^^,^^43^ In addition, bergenin has been found to overcome the resistance of cancer cells to radiotherapy.^41^ These findings suggest that Tan IIA and bergenin have potential as antitumor agents for the prevention and treatment of OSCC. In addition, it has been noted that GLUT-1 inhibitors (Fasentin, STF-31, and WZB117) can inhibit cancer cell activity.^222^^,^^223^ Interestingly, in OSCC, GLUT3 expression can be compensatorily upregulated when GLUT1 is inhibited, suggesting that targeting GLUT3 may be a new approach to the treatment of OSCC.^224^ Overall, GLUTs have not been sufficiently studied in OSCC. Studies have reported that MCTs can be inhibited by various compounds via nonspecific binding, resulting in decreased activity or inactivation.^225^ Specifically, 4′-diisothiocyano-2,2′-stilbenedisulphonate (DIDS) affects the activities of MCT1 and MCT2.^225^ Since 2007, there has been a growing interest in the immunosuppressive target MCT1, and this interest has led to the identification of compounds AZD3965 and ARC155858 as inhibitors of MCT1 and MCT2.^44^ However, these compounds do not inhibit MCT4.^226^^,^^227^ Among the latest advances, BAY-8002, a novel MCT1 inhibitor developed in recent years, is significantly more selective for MCT1 than other MCT proteins.^228^

In HNSCC, obesity, lipid metabolism-related genes, and poor prognosis of HNSCC patients are significantly correlated.^75^ Diet-induced obesity can significantly increase the risk of OSCC.^229^ Dietary interventions to mitigate the risk of OSCC by reducing fat intake and controlling obesity are a potential avenue for preventive measures. Statins are commonly used to interfere with the lipid metabolism and cholesterol synthesis in cancer patients. The inhibitory effects of pitavastatin and simvastatin on OSCC proliferation and invasion have been elucidated in several studies. Pitavastatin, acting as a radiosensitizer, enhances the efficacy of radiotherapy in HNSCC by inhibiting the mevalonate pathway, which plays a key role in tumor cell survival and DNA repair.^230^ The synergistic efficacy of pitavastatin and camatinib, a MET-specific inhibitor, further emphasizes the potential of combination therapies in OSCC treatment.^102^^,^^231^, ^232^, ^233^ FASN has emerged as a promising target for OSCC treatment, and inhibition of FASN has showed the ability to attenuate tumor size and reduce proliferation capacity.^86^^,^^234^ This inhibition enhances the sensitivity of OSCC cells to conventional chemotherapeutic agents such as cisplatin and paclitaxel.^235^ The FASN inhibitor TVB-3166 showed promising anti-proliferative effects on OSCC cell lines, although further preclinical studies are needed. Despite the potent anti-tumor properties of certain FASN inhibitors, clinical evaluation has encountered obstacles related to drug metabolism, such as problems with orlistat.^236^ Overcoming these challenges is essential to translate the therapeutic potential of FASN inhibitors into clinical practice.

Targeting amino acid metabolism is also a potential strategy for the treatment of HNSCC.^237^, ^238^, ^239^, ^240^, ^241^ Arginine and glutamine are crucial for energy metabolism in OSCC and are significant targets. ASCT2 is a protein that transports glutamine across the cell membrane and plays a critical role in several types of tumors.^120^^,^^242^, ^243^, ^244^ A recent study found that the knockdown of ASCT2 inhibited the growth of OSCC *in vivo*, suggesting that the knockdown of ASCT2 has anti-tumor properties.^118^ This could be a potential therapeutic target for the treatment of OSCC. The anti-EGFR drug cetuximab is commonly used clinically to improve the prognosis of HNSCC patients.^245^ In addition, studies have shown that a combination regimen by inhibition of glutamine uptake combined with cetuximab is expected to improve the prognosis of HNSCC patients.^215^ Arginine deprivation therapy is a targeted metabolic therapy that has made considerable progress in recent years, and its principle of action is to prevent cancer cells from synthesizing new arginine.^246^ Canavanine is a natural arginine analogue that has been shown to inhibit cancer cells, leading to cell death.^247^, ^248^, ^249^, ^250^ It has been suggested that combining arginine deprivation therapy with low concentrations of canavanine produces a more effective synergistic effect and promotes the radiosensitization of HNSCC.^246^

More and more cancer treatments are turning to immunotherapy, commonly involving PD-L1/PD-1 inhibitors. In locally advanced HNSCC patients with high PD-L1 expression, the tumor microenvironment exhibits an active immune response; however, the therapeutic efficacy of adjuvant radiotherapy remains limited. This may be due to radiotherapy suppressing immune defense mechanisms during treatment. In contrast, in locally advanced HNSCC patients with low PD-L1 expression, the lack of a prominent immunosuppressive environment allows radiotherapy to achieve better therapeutic outcomes. Therefore, for PD-L1 high-expressing patients, adjuvant immune checkpoint inhibitors may offer a more effective alternative therapeutic strategy.^251^ In HNSCC, it is essential to note that a crucial prerequisite for the use of PD-L1/PD-1 inhibitors in immunotherapy is the integrity of MHC-I APM and the substantial activation of MHC-I.^252^ To achieve this, MHC-I activation can be facilitated by activating INF and inhibiting PI3K, EGFR, and SHP-2. This leads to the mechanism behind PD-L1/PD-1 therapy, which aims to modify the interaction between immune cells and cancer cells so that the immune cells re-recognize the cancer cells and activate T-cell responses. Additionally, anti-PD-1 treatment is known for its high efficiency, low toxicity, and minimal adverse reactions, thus enhancing the survival and prognosis of OSCC patients.^253^ Furthermore, the synergistic combination therapy using the anti-PD-1 antibody camrelizumab and the VEGFR inhibitor apatinib has been well-received in OSCC, showing good tolerability, no serious adverse effects, and less than 10% of remaining live tumor cells.^254^ This opens the door for further investigation into the efficacy of anti-PD-1 and anti-VEGFR combination therapy trials. When considering chemotherapy combined with immunotherapy for HNSCC patients, it is critical to recognize that different treatments may significantly affect T cell activity, tumor sensitivity, and overall patient response. This requires a detailed exploration of the optimal timing for chemotherapy and immunotherapy to achieve the best therapeutic outcomes.^255^ Additionally, other clinical trials aim to combine immunotherapeutic drugs with other therapeutic approaches, including additional immunosuppressants, HPV vaccines, and patient-specific tumor vaccines, to take advantage of the synergistic effects of diverse treatments to fight cancer more effectively.^256^

## Conclusion and perspectives

Immunotherapy and various targeted therapies have evolved rapidly over the years to provide substantial benefits to HNSCC patients. Among them, targeted therapies against immune cells and regulators of various metabolites in tumor metabolic reprogramming are in progress, especially against lactate, mTOR, and HIF1. However, despite these advancements, immunotherapy and several targeted therapies have not yet achieved the expected effects in many patients. To address this issue, the descriptions of various energy metabolism in HNSCC contribute to uncovering new therapeutic possibilities. These include specifically targeting key molecules or proteins in abnormal energy metabolism pathways to enhance the clinical treatment efficacy for HNSCC patients.

## CRediT authorship contribution statement

**Haofan Li:** Writing – review & editing, Writing – original draft. **Qiu Peng:** Writing – review & editing, Writing – original draft. **Linda Oyang:** Writing – review & editing, Writing – original draft. **Wenjuan Yang:** Writing – review & editing, Writing – original draft. **Shizhen Li:** Writing – review & editing, Writing – original draft. **Yaqian Han:** Writing – review & editing, Writing – original draft. **Mingjing Peng:** Writing – review & editing, Writing – original draft, Conceptualization. **Shiming Tan:** Writing – review & editing, Writing – original draft. **Longzheng Xia:** Writing – review & editing, Writing – original draft. **Jinguan Lin:** Writing – review & editing, Writing – original draft. **Xuemeng Xu:** Writing – review & editing, Writing – original draft. **Nayiyuan Wu:** Writing – review & editing, Writing – original draft. **Yanyan Tang:** Writing – review & editing, Writing – original draft. **Xia Luo:** Writing – review & editing, Writing – original draft. **Xianjie Jiang:** Writing – review & editing, Writing – original draft. **Qianjin Liao:** Writing – review & editing, Writing – original draft. **Yujuan Zhou:** Writing – review & editing, Writing – original draft.

## Funding

This work was supported in part by grants from the following sources: the National Natural Science Foundation of China (No. 82302987, 82303534, 82203233, 82202966, 82173142), the Natural Science Foundation of Hunan Province, China (No. 2023JJ60469, 2023JJ40413, 2023JJ30372, 2023JJ30375, 2023JJ40417, 2020JJ5336), the Research Project of Health Commission of Hunan Province, China (No. Z2023086, R2023093, R2023040, 202203034978, 202202055318, 202203231032, 202109031837, 202109032010, 20201020), the Science and Technology Innovation Program of Hunan Province, China (No. 2023ZJ1122, 2023RC3199, 2023SK4034, 2023RC1073, 2022SK2051), the Hunan Provincial Science and Technology Department of China (No. 2020TP1018), the Changsha Science and Technology Board of China (No. kh2201054), the Ascend Foundation of National Cancer Center, China (No. NCC201909B06), and the Hunan Cancer Hospital Climb Plan, China (No. ZX2020001-3, YF2020002).

## Conflict of interests

The authors declared no competing interests.
